# The volume of outpatient office visits did not increase for specialties that were more likely to adopt telehealth

**DOI:** 10.1093/haschl/qxaf227

**Published:** 2025-11-25

**Authors:** James D Lee, Elena Chun, Chiang-Hua Chang, Hechuan Hou, Terrence Liu, Rodney L Dunn, Jeffrey S McCullough, Michael P Thompson, Chad Ellimoottil

**Affiliations:** Department of Internal Medicine, University of Michigan, Ann Arbor, MI 48109, United States; Institute for Healthcare Policy & Innovation, University of Michigan, Ann Arbor, MI 48109, United States; Institute for Healthcare Policy & Innovation, University of Michigan, Ann Arbor, MI 48109, United States; Department of Urology, University of Michigan, Ann Arbor, MI 48109, United States; Institute for Healthcare Policy & Innovation, University of Michigan, Ann Arbor, MI 48109, United States; Department of Cardiac Surgery, University of Michigan, Ann Arbor, MI 48109, United States; Institute for Healthcare Policy & Innovation, University of Michigan, Ann Arbor, MI 48109, United States; Department of Cardiac Surgery, University of Michigan, Ann Arbor, MI 48109, United States; Department of Internal Medicine, University of Michigan, Ann Arbor, MI 48109, United States; Institute for Healthcare Policy & Innovation, University of Michigan, Ann Arbor, MI 48109, United States; Institute for Healthcare Policy & Innovation, University of Michigan, Ann Arbor, MI 48109, United States; Department of Urology, University of Michigan, Ann Arbor, MI 48109, United States; Institute for Healthcare Policy & Innovation, University of Michigan, Ann Arbor, MI 48109, United States; Department of Health Management and Policy, University of Michigan, Ann Arbor, MI 48109, United States; Institute for Healthcare Policy & Innovation, University of Michigan, Ann Arbor, MI 48109, United States; Department of Cardiac Surgery, University of Michigan, Ann Arbor, MI 48109, United States; Institute for Healthcare Policy & Innovation, University of Michigan, Ann Arbor, MI 48109, United States; Department of Urology, University of Michigan, Ann Arbor, MI 48109, United States

## Introduction

Telehealth use expanded rapidly during the COVID-19 pandemic and has become an integral part of healthcare delivery. However, concerns persist that increased telehealth availability may contribute to higher healthcare utilization without significantly improving quality. As federal policymakers deliberate permanent telehealth coverage policies, it is critical to evaluate whether expanded telehealth use has increased overall utilization.

Several studies have examined the association between expanded telehealth use and healthcare utilization in Medicare fee-for-service (FFS) populations, with mixed findings. Analyses by the Medicare Payment Advisory Commission and the American Institutes for Research found that hospital service areas with high telehealth use saw higher total clinician encounters compared to those with low telehealth use.^1^ Another study comparing health systems with varying levels of telehealth use found a small increase in total outpatient office visits among high telehealth-use systems.^2^ However, disentangling the impact of telehealth adoption from underlying health system or geographic factors remains a key challenge.

Our objective was to assess whether increased telehealth adoption was associated with changes in overall outpatient office visits among Medicare beneficiaries by comparing specialties with low, medium, and high telehealth adoption.

## Methods

We analysed 100% of Medicare FFS claims from Carrier and Outpatient files from January 2019 to June 2024. We included FFS beneficiaries aged 65 and older with Part A and Part B coverage. We excluded counties with fewer than 10 FFS beneficiaries or fewer than 10 outpatient office visits per month per specialty.

We identified all outpatient evaluation and management visits using Berenson-Eggers Type of Service codes. Telehealth visits were classified based on Medicare's list of eligible telehealth services and the corresponding modifier or place of service codes for each study year (Appendix Table S1).

Our main exposure was how likely specialties were to adopt telehealth, categorized into low, medium, and high telehealth groups (Appendix Table S2). The low telehealth group included otolaryngology, ophthalmology, orthopaedic surgery, sports medicine, plastic and reconstructive surgery, hand surgery, podiatry, and maxillofacial surgery. The medium telehealth group included primary care specialties and additional medical and surgical subspecialties. The high telehealth group comprised behavioural health specialties. The primary outcome was total outpatient office visits, including telehealth and in-person visits.

We conducted a difference-in-differences (DID) analysis comparing total outpatient office visit volumes pre- (January 2019–February 2020) and post-pandemic (January 2021–June 2024) periods across telehealth groups, using the low telehealth group as the control. The analysis included county fixed effects and adjusted for county-level demographics and comorbidities. We visually assessed that pre-pandemic trends in our outcome were parallel across the specialty groups. A formal test indicated that while trends were similar, small differences in slopes existed between groups (Appendix Figure S1).

## Results

A total of 60 531 036 patients were included (Appendix Table S3), with 538 801 822 outpatient office visits. In the post-pandemic period, telehealth comprised 5.3%, 9.1%, and 43.8% of total outpatient office visits for the low, medium, and high telehealth groups, respectively (Appendix Figure S2).

Despite variation in telehealth adoption post-pandemic, all 3 groups experienced declines in predicted number of total outpatient office visits: a 14% decrease for the low telehealth group (from 12 588 to 10 831 predicted visits per month per county), a 17% decrease for the medium telehealth group (from 18 873 to 15 733), and an 18% decrease for the high telehealth group (from 3264 to 2682) (Table 1). Compared to the low telehealth group, both medium (incidence rate ratio: 0.96, 95% CI: 0.96-0.97) and high (incidence rate ratio: 0.91, 95% CI: 0.90-0.92) telehealth groups saw significantly larger declines post-pandemic (Table 1).

**Table 1.** Difference-in-differences model estimates and predicted total outpatient office visits per county-month by telehealth group.

Panel A: Different-in-differences model estimates				
Variables	Log-incidence rate ratio	95% CI	Incidence rate ratio	95% CI
Post-pandemic period	−0.04	(−0.04, −0.03)	0.96^a^	(0.96, 0.97)
Medium telehealth group	0.42	(0.40, 0.43)	1.52^a^	(1.49, 1.54)
High telehealth group	−1.50	(−1.55, −1.45)	0.22^a^	(0.21, 0.23)
DID Estimator—medium vs low	−0.04	(−0.04, −0.03)	0.96^a^	(0.96, 0.97)
DID Estimator—high vs low	−0.09	(−0.10, −0.08)	0.91^a^	(0.90, 0.92)

Panel B: Predicted total outpatient office visits per county-month			
Telehealth groups	Pre-pandemic period	Post-pandemic period	Relative change
Low telehealth	12 588	10 831	−14%
Medium telehealth	18 873	15 733	−17%
High telehealth	3264	2682	−18%

Authors' analysis of a 100% sample of Medicare fee-for-service beneficiaries from January 1, 2019, through June 30, 2024.

Low telehealth group includes otolaryngology, ophthalmology, orthopaedic surgery, sports medicine, plastic and reconstructive surgery, hand surgery, podiatry, and maxillofacial surgery. Medium telehealth group includes general practice, family practice, internal medicine, osteopathic manipulative therapy, hospice and palliative care, geriatric medicine, preventive medicine, gastroenterology, pulmonary disease, nephrology, infectious disease, endocrinology, medical oncology, neurosurgery, thoracic surgery, and surgical oncology. High telehealth group includes psychiatry, general psychiatry, psychology, clinical psychology, and neuropsychiatry.

Pre-pandemic period was classified as January 2019–February 2020, and post-pandemic period was classified as January 2021–June 2024.

Model adjusted for county-level average patient age, sex distribution, racial composition, average Hierarchical Chronic Condition score, proportion of dual eligible beneficiaries, and proportion of patients in fee-for-service (FFS). The model includes county fixed effects. Counties with fewer than 10 fee-for-service beneficiaries or fewer than 10 outpatient office visits per month per specialty group were excluded.

^a^ *P* < 0.0001.

## Discussion

Despite variation in telehealth adoption, total outpatient office visits remained stable or declined slightly in groups with higher telehealth use compared to the low telehealth group.

Our findings align with some studies reporting minimal changes in overall outpatient volume after telehealth expansion. An analysis of primary care encounters across 3 large healthcare systems found little difference in total outpatient visits despite increased telehealth utilization.^3^ Similarly, a study of commercially insured patients found that the overall number of outpatient office visits in the 30 days following hospital discharge did not change significantly, despite a rapid increase in telehealth use.^4^

Several factors may explain why total outpatient office visit volume did not increase despite expanded use of telehealth. First, provider capacity likely remained a limiting factor, as most clinicians operate on fixed schedules, and telehealth did not necessarily expand appointment availability. Second, the ongoing shortage of primary care and mental health providers may have constrained the ability to accommodate increased demand post-pandemic, even with telehealth.^5^ Third, some patients may have used telehealth selectively for convenience rather than increasing their overall demand for care.

Notably, telehealth accounted for 43.8% of outpatient behavioural health visits post-pandemic. Given the substantial reliance on telehealth for mental health care and barriers older adults face accessing in-person services, disruption to telehealth coverage may limit access to mental health services for Medicare beneficiaries.

Our study examined telehealth's impact using specialty-level variation in telehealth adoption, complementing prior work that focused on practice- or geographic-level comparisons. This approach mitigates some methodological limitations from earlier work where practice- or geographical-level factors could confound the relationship between telehealth use and overall utilization.

Some limitations of the study include a focus on Medicare FFS beneficiaries and potential residual time-varying confounders. We also did not evaluate the quality of care between in-person and telehealth visits or healthcare utilization beyond outpatient office visits. Nevertheless, understanding telehealth's impact on Medicare FFS outpatient office visit utilization, which constitutes a significant portion of Medicare spending, is essential for guiding policy decisions. Lastly, although minor differences existed in pre-pandemic outpatient office visit trends across groups, these were small and unlikely to meaningfully affect our conclusion.

Widespread telehealth adoption post-pandemic does not appear to be associated with an increase in total outpatient office visit utilization among Medicare FFS beneficiaries. Instead, telehealth visits appear largely to have served as a substitute for in-person visits, supporting telehealth's role as an alternative care delivery model without substantially increasing outpatient office visit volume.

## Supplementary Material

qxaf227_Supplementary_Data

## References

[1] Saharkhiz M, Rao T, Parker-Lue S, Borelli S, Johnson K, Cataife G. Telehealth expansion and Medicare beneficiaries' care quality and access. JAMA Netw Open. 2024;7(5):e2411006. 10.1001/jamanetworkopen.2024.11006 38739388 PMC11091757

[2] Nakamoto CH, Cutler DM, Beaulieu ND, Uscher-Pines L, Mehrotra A. The impact of telemedicine on Medicare utilization, spending, and quality, 2019–22. Health Aff (Millwood). 2024;43(5):691–700. 10.1377/hlthaff.2023.01142 38630943 PMC13400057

[3] Dixit RA, Ratwani RM, Bishop JA, et al The impact of expanded telehealth availability on primary care utilization. NPJ Digit Med. 2022;5(1):1–3. 10.1038/s41746-022-00685-8 36085158 PMC9462602

[4] Bressman E, Russo A, Werner RM. Trends in outpatient care and use of telemedicine after hospital discharge in a large commercially insured population. JAMA Health Forum. 2021;2(11):e213685. 10.1001/jamahealthforum.2021.3685 35977266 PMC8796902

[5] McBain RK, Schuler MS, Breslau J, Kofner A, Wang L, Cantor JH. Telehealth availability for mental health care during and after the COVID-19 public health emergency. JAMA Netw Open. 2024;7(7):e2420853. 10.1001/jamanetworkopen.2024.20853 38985472 PMC11238022

