# Supplementary material for: The volume of outpatient office visits did not increase for specialties that were more likely to adopt telehealth
Source: Health Aff Sch. 2025 Nov 25;3(12):qxaf227. doi: 10.1093/haschl/qxaf227 (PMC12672024; doi:10.1093/haschl/qxaf227)
Supplement: qxaf227_Supplementary_Data [file qxaf227_supplementary_data.zip › Supplemental Material (final edits).docx]

**APPENDIX**

**Table of Contents**

Appendix Table A1 - Codes and modifiers used to identify telehealth services.

Appendix Table A2 - Specialty codes used to identify high, medium, and low telehealth groups.

Appendix Figure A1 - Event study graph for high and medium telehealth groups pre- and post-pandemic.

Appendix Table A3 - Patient characteristics of outpatient office visits for low, medium, and high telehealth groups in Medicare fee-for-service, January 2019–June 2024.

Appendix Figure A2 - Monthly trends in percentage of telehealth office visits for low, medium, and high telehealth groups, January 2019–June 2024.

Appendix Figure A3 - Monthly trends in total outpatient office visits per 1,000 beneficiaries for low, medium, and high telehealth groups, January 2019–June 2024.

Appendix Figure A4 - Monthly trends in percentage of telehealth office visits by specialty in Medicare fee-for-service, January 2019–June 2024.

Appendix Figure A5 - Monthly trends in total outpatient office visits per 1,000 beneficiaries by specialty in Medicare fee-for-service, January 2019–June 2024.

Appendix Table A4 - Difference-in-differences models of specialty group comparisons for rates of outpatient office visits per county-month.

**Appendix Table A1. Codes and modifiers used to identify telehealth services.**

| **Type of telehealth** | **Codes** |
| --- | --- |
| Modifier Codes/Place of Service | Modifier: G0 (zero) or GQ or GT or FR or FQ or 93, or 95 or |
|  | Place of service: 02 or 10 |
| 1. Video+Audio or Audio only | (On top of having modifier/POS) HCPCS codes in CMS annual telehealth list 2019-2024 |
| 2. Audio Only | (On top of having modifier/POS) HCPCS codes in CMS annual telehealth list 2019-2024 with Audio only |
| Phone | 99441, 99442, 99443 |
| Online digital E&M for established patients | 99421, 99422, 99423, G2061/98970, G2062/98971, G2063/98972 |
| Remote physiologic monitoring | 99453, 99454, 99457, 99458, 99091 |
| Remote therapeutic monitoring | 98975, 98976, 98977, 98980, 98981 |
| Interprofessional internet consultation | 99446, 99447, 99448, 99449, 99451, 99452 |
| Virtual check-ins | G2010/G2250, G2012/G2251, G2252 |
| Safety-net providers | G0071, G2025 |
| Other | G0459, 98966, 98967, 98968, 98969, 99444, 0448T, 994X0, G0320, G0321, G0322 |
| Total telehealth visits | Any above |

NOTES Obtained from Centers for Medicare & Medicaid Services. List of Telehealth Services. Available from: <https://www.cms.gov/medicare/coverage/telehealth/list-services>

**Appendix Table A2. Specialty codes used to identify high, medium, and low telehealth groups.**

| **Level of Telehealth Use** | **Specialty Description** | **Specialty Code** |
| --- | --- | --- |
| High | Behavioral health |  |
|  | Psychiatry | 26 |
|  | General psychiatry | 27 |
|  | Psychology | 62 |
|  | Clinical psychology | 68 |
|  | Neuropsychiatry | 86 |
| Medium | Primary Care |  |
|  | FQHC/RHC | n/a |
|  | General practice | 1 |
|  | Family practice | 8 |
|  | Internal medicine | 11 |
|  | Hospice and palliative care | 17 |
|  | Geriatric medicine | 38 |
|  | Preventive medicine | 84 |
|  | Medical subspecialties |  |
|  | Gastroenterology | 10 |
|  | Pulmonary disease | 29 |
|  | Nephrology | 39 |
|  | Infectious disease | 44 |
|  | Endocrinology | 46 |
|  | Medical oncology | 90 |
|  | Surgical subspecialties |  |
|  | Neurosurgery | 14 |
|  | Thoracic surgery | 33 |
|  | Surgical oncology | 91 |
| Low | Otolaryngology | 4 |
|  | Ophthalmology | 18 |
|  | Orthopedic surgery | 20 |
|  | Sports medicine | 23 |
|  | Plastic and reconstructive surgery | 24 |
|  | Hand surgery | 40 |
|  | Podiatry | 48 |
|  | Maxillofacial surgery | 85 |

NOTES We initially selected three specialties—orthopedic surgery, primary care, and behavioral health—a priori and hypothesized that they would have low, medium, and high telehealth adoption, respectively. Using Provider Specialty Codes, we grouped specialties into the low, medium, and high telehealth groups based on clinical similarities in visit characteristics to above specialties and prior literature analyzing telehealth utilization.^1–4^

*1. Chao GF, Li KY, Zhu Z, et al. Use of Telehealth by Surgical Specialties During the COVID-19 Pandemic. JAMA Surg. 2021;156(7):620-626. doi:10.1001/jamasurg.2021.0979*

*2. Patel SY, Mehrotra A, Huskamp HA, Uscher-Pines L, Ganguli I, Barnett ML. Variation In Telemedicine Use And Outpatient Care During The COVID-19 Pandemic In The United States. Health Aff (Millwood). 2021;40(2):349-358. doi:10.1377/hlthaff.2020.01786*

*3. Hsiao V, Chandereng T, Huebner JA, et al. Telemedicine Use across Medical Specialties and Diagnoses. Appl Clin Inform. 2023;14:172-184. doi:10.1055/s-0043-1762595*

*4. Yu J, Civelek Y, Casalino LP, et al. Telehealth Delivery Differs Significantly By Physician And Practice Characteristics. Health Aff (Millwood). 2024;43(9):1311-1318. doi:10.1377/hlthaff.2024.00052*

**Appendix Figure A1. Event study graph for high and medium telehealth groups pre- and post-pandemic.**

*Panel 1: Relative per month per county change in outpatient office visits, expressed as log-incidence rate ratios with 95% confidence intervals, for high telehealth group compared to low telehealth group.*

*Panel 2: Relative per month per county change in outpatient office visits, expressed as log-incidence rate ratios with 95% confidence intervals, for medium telehealth group compared to low telehealth group.*

SOURCE Authors’ analysis of a 100 percent sample of Medicare fee-for-service beneficiaries from January 1, 2019 through June 30, 2024.

NOTES Low telehealth group includes otolaryngology, ophthalmology, orthopedic surgery, sports medicine, plastic and reconstructive surgery, hand surgery, podiatry, and maxillofacial surgery. Medium telehealth group includes general practice, family practice, internal medicine, osteopathic manipulative therapy, hospice and palliative care, geriatric medicine, preventive medicine, gastroenterology, pulmonary disease, nephrology, infectious disease, endocrinology, medical oncology, neurosurgery, thoracic surgery, and surgical oncology. High telehealth group includes psychiatry, general psychiatry, psychology, clinical psychology, and neuropsychiatry.

Model adjusted for county-level average patient age, sex distribution, racial composition, average Hierarchical Chronic Condition score, proportion of dual eligible beneficiaries, and proportion of patients in fee-for-service (FFS). Model includes county fixed effects. Counties with fewer than 10 fee-for-service beneficiaries or fewer than 10 office visits per month per specialty group were excluded.

**Appendix Table A3. Patient characteristics of outpatient office visits for low, medium, and high telehealth groups in Medicare fee-for-service, January 2019 - June 2024.**

| **Characteristic** | **Low**  **telehealth group** | **Medium**  **telehealth group** | **High**  **telehealth group** |
| --- | --- | --- | --- |
| Beneficiary count | 27,784,361 | 28,374,123 | 4,372,552 |
| Age, mean (sd) | 72.1 (10.1) | 71.6 (10.9) | 67.6 (15.1) |
| Hierarchical Condition Category score, median (IQR) | 0.294 (0-0.692) | 0.318 (0-0.817) | 0.608 (0.298-1.25) |
| Dual Eligibility, n (%) | 3,243,724 (11.7%) | 3,837,428 (13.5%) | 1,306,703 (29.9%) |
| Male, n (%) | 11,871,183 (42.7%) | 12,432,345 (43.8%) | 1,777,712 (40.7%) |
| Race, n (%) |  |  |  |
| White | 22,612,088 (81.4%) | 22,509,718 (79.3%) | 3,437,671 (78.6%) |
| Black | 2,038,636 (7.3%) | 2,412,242 (8.5%) | 432,819 (9.9%) |
| Hispanic | 1,419,725 (5.1%) | 1,591,250 (5.6%) | 272,665 (6.2%) |
| Asian | 747,503 (2.7%) | 862,410 (3.0%) | 84,929 (1.9%) |
| Other | 344,867 (1.2%) | 362,888 (1.3%) | 50,143 (1.1%) |
| Unknown | 621,542 (2.2%) | 635,615 (2.2%) | 94,325 (2.2%) |

SOURCE Authors’ analysis of a 100 percent sample of Medicare fee-for-service beneficiaries from January 1, 2019 through June 30, 2024.

NOTES Low telehealth group includes otolaryngology, ophthalmology, orthopedic surgery, sports medicine, plastic and reconstructive surgery, hand surgery, podiatry, and maxillofacial surgery. Medium telehealth group includes general practice, family practice, internal medicine, osteopathic manipulative therapy, hospice and palliative care, geriatric medicine, preventive medicine, gastroenterology, pulmonary disease, nephrology, infectious disease, endocrinology, medical oncology, neurosurgery, thoracic surgery, and surgical oncology. High telehealth group includes psychiatry, general psychiatry, psychology, clinical psychology, and neuropsychiatry. Counties with fewer than 10 fee-for-service beneficiaries or fewer than 10 office visits per month per specialty group were excluded.

**Appendix Figure A2. Monthly trends in percentage of telehealth office visits for low, medium, and high telehealth groups, January 2019 – June 2024.**


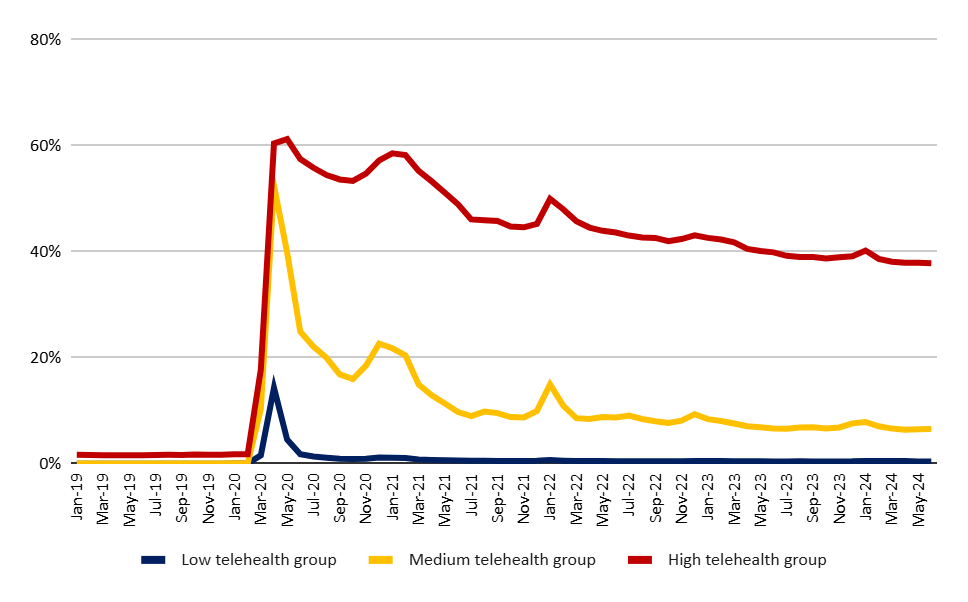


SOURCE Authors’ analysis of a 100 percent sample of Medicare fee-for-service beneficiaries from January 1, 2019 through June 30, 2024.

NOTES Low telehealth group includes otolaryngology, ophthalmology, orthopedic surgery, sports medicine, plastic and reconstructive surgery, hand surgery, podiatry, and maxillofacial surgery. Medium telehealth group includes general practice, family practice, internal medicine, osteopathic manipulative therapy, hospice and palliative care, geriatric medicine, preventive medicine, gastroenterology, pulmonary disease, nephrology, infectious disease, endocrinology, medical oncology, neurosurgery, thoracic surgery, and surgical oncology. High telehealth group includes psychiatry, general psychiatry, psychology, clinical psychology, and neuropsychiatry. Counties with fewer than 10 fee-for-service beneficiaries or fewer than 10 office visits per month per specialty were excluded.

**Appendix Figure A3. Monthly trends in total outpatient office visits per 1,000 beneficiaries for low, medium, and high telehealth groups, January 2019 - June 2024.**


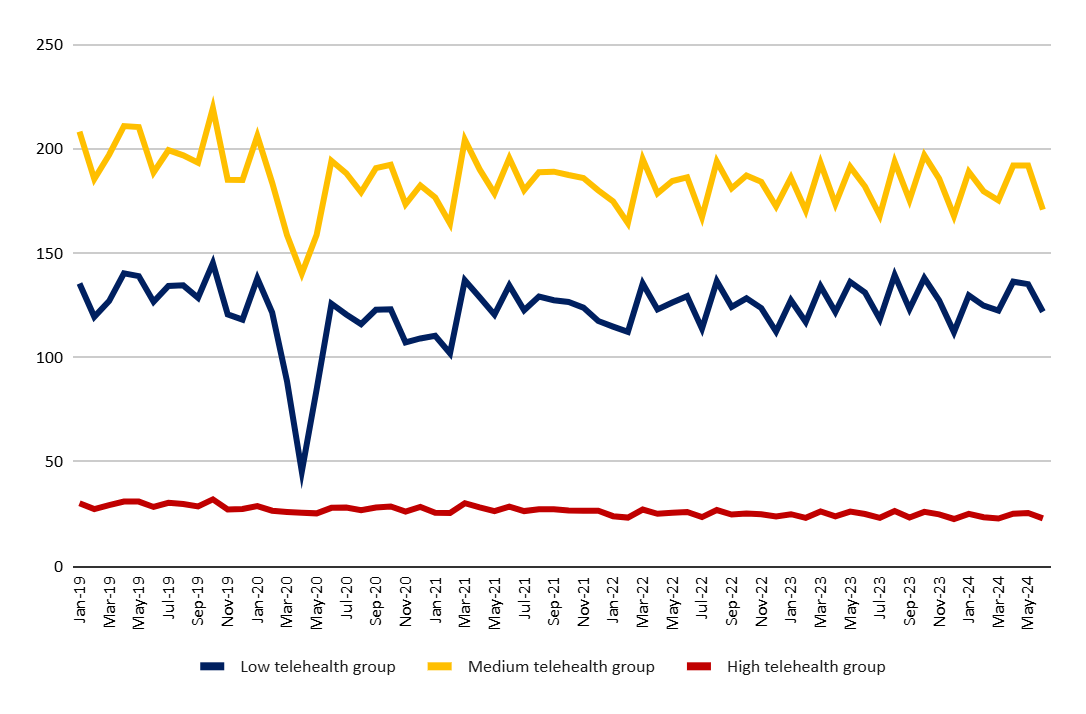


SOURCE Authors’ analysis of a 100 percent sample of Medicare fee-for-service beneficiaries from January 1, 2019 through June 30, 2024.

NOTES Low telehealth group includes otolaryngology, ophthalmology, orthopedic surgery, sports medicine, plastic and reconstructive surgery, hand surgery, podiatry, and maxillofacial surgery. Medium telehealth group includes general practice, family practice, internal medicine, osteopathic manipulative therapy, hospice and palliative care, geriatric medicine, preventive medicine, gastroenterology, pulmonary disease, nephrology, infectious disease, endocrinology, medical oncology, neurosurgery, thoracic surgery, and surgical oncology. High telehealth group includes psychiatry, general psychiatry, psychology, clinical psychology, and neuropsychiatry. Counties with fewer than 10 fee-for-service beneficiaries or fewer than 10 office visits per month per specialty were excluded.

**Appendix Figure A4. Monthly trends in percentage of telehealth office visits by specialty in Medicare fee-for-service, January 2019 - June 2024.**

SOURCE Authors’ analysis of a 100 percent sample of Medicare fee-for-service beneficiaries from January 1, 2019 through June 30, 2024.

NOTES All counties with fewer than 10 fee-for-service beneficiaries or fewer than 10 office visits per month per specialty were excluded.

**Appendix Figure A5. Monthly trends in total outpatient office visits per 1,000 beneficiaries by specialty in Medicare fee-for-service, January 2019 - June 2024.**

SOURCE Authors’ analysis of a 100 percent sample of Medicare fee-for-service beneficiaries from January 1, 2019 through June 30, 2024.

NOTES All counties with fewer than 10 fee-for-service beneficiaries or fewer than 10 office visits per month per specialty were excluded.

**Appendix Table A4. Difference-in-differences models of different specialty group comparisons for rates of outpatient office visits per county-month.**

| *Model 1. Low telehealth - control (orthopedic surgery) vs. Medium telehealth (primary care) vs. High telehealth (behavioral health)* | | |
| --- | --- | --- |
| **Variables** | **Log-Incidence Rate Ratio** | **95% CI** |
| Post-Pandemic Period | -0.004 | (-0.02, 0.01) |
| Medium Telehealth | 1.56 | (1.53, 1.60) |
| High Telehealth | -0.32 | (-0.39, -0.25) |
| DID Estimator - Medium Telehealth vs. Low Telehealth | -0.08 | (-0.09, -0.06) |
| DID Estimator - High Telehealth vs. Low Telehealth | -0.04 | (-0.05, -0.03) |
|  |  |  |
| *Model 2. Low telehealth - control (composite specialties) vs. Medium telehealth (primary care) vs. High telehealth (behavioral health)* | | |
| **Variables** | **Log-Incidence Rate Ratio** | **95% CI** |
| Post-Pandemic Period | -0.03 | (-0.04, -0.02) |
| Medium Telehealth | -0.005 | (-0.03, 0.02) |
| High Telehealth | -1.50 | (-1.54, -1.45) |
| DID Estimator - Medium Telehealth vs. Low Telehealth | -0.06 | (-0.07, -0.05) |
| DID Estimator - High Telehealth vs. Low Telehealth | -0.09 | (-0.10, -0.08) |
|  |  |  |
| *Model 3. Low telehealth - control (composite specialties) vs. Medium telehealth (primary care and other medical and surgical specialties) vs. High telehealth (behavioral health)* | | |
| **Variables** | **Log-Incidence Rate Ratio** | **95% CI** |
| Post-Pandemic Period | -0.04 | (-0.04, -0.03) |
| Medium Telehealth | 0.42 | (0.40, 0.43) |
| High Telehealth | -1.50 | (-1.55, -1.45) |
| DID Estimator - Medium Telehealth vs. Low Telehealth | -0.04 | (-0.04, -0.03) |
| DID Estimator - High Telehealth vs. Low Telehealth | -0.09 | (-0.10, -0.08) |
|  |  |  |
| *Model 4. Model 3 without covariates* | | |
| **Variables** | **Log-Incidence Rate Ratio** | **95% CI** |
| Post-Pandemic Period | -0.08 | (-0.09, -0.08) |
| Medium Telehealth | 0.42 | (0.40, 0.43) |
| High Telehealth | -1.50 | (-1.54, -1.45) |
| DID Estimator - Medium Telehealth vs. Low Telehealth | -0.04 | (-0.05, -0.03) |
| DID Estimator - High Telehealth vs. Low Telehealth | -0.09 | (-0.10, -0.08) |
